# Supplementary figures and images for: A comprehensive performance analysis of sequence-based within-sample testing NIPT methods
Source: PLoS One. 2023 Apr 14;18(4):e0284493. doi: 10.1371/journal.pone.0284493 (PMC10104307; doi:10.1371/journal.pone.0284493)

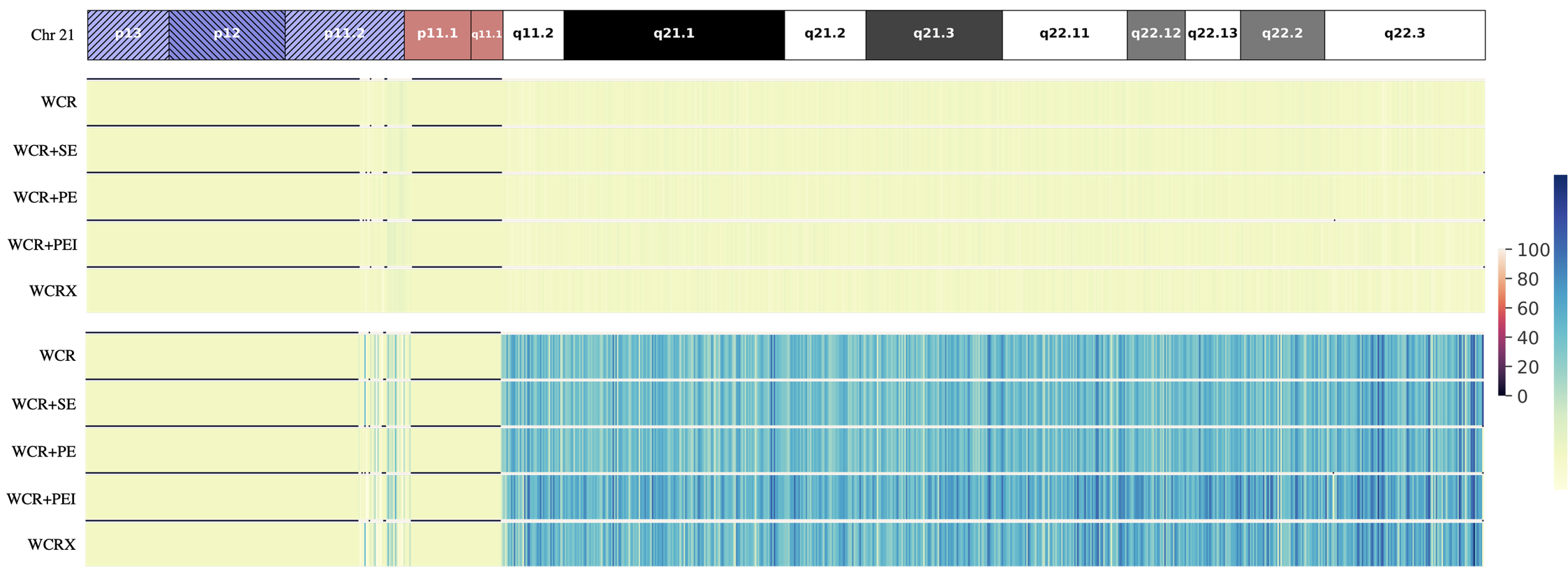

Supplement: S1 Fig — Heatmap of the summed per bin Z-scores across all negative (top) and all T21 positive (bottom) samples at a 50 kb bin scale for chromosome 21 and all different Wisecondor-based methods. The line above each method’s heatmap corresponds to the average number of selected reference bins for each bin of that method (black denoting that no similar reference bins are found and consequently these bins are excluded). (TIF) [file pone.0284493.s001.tif]

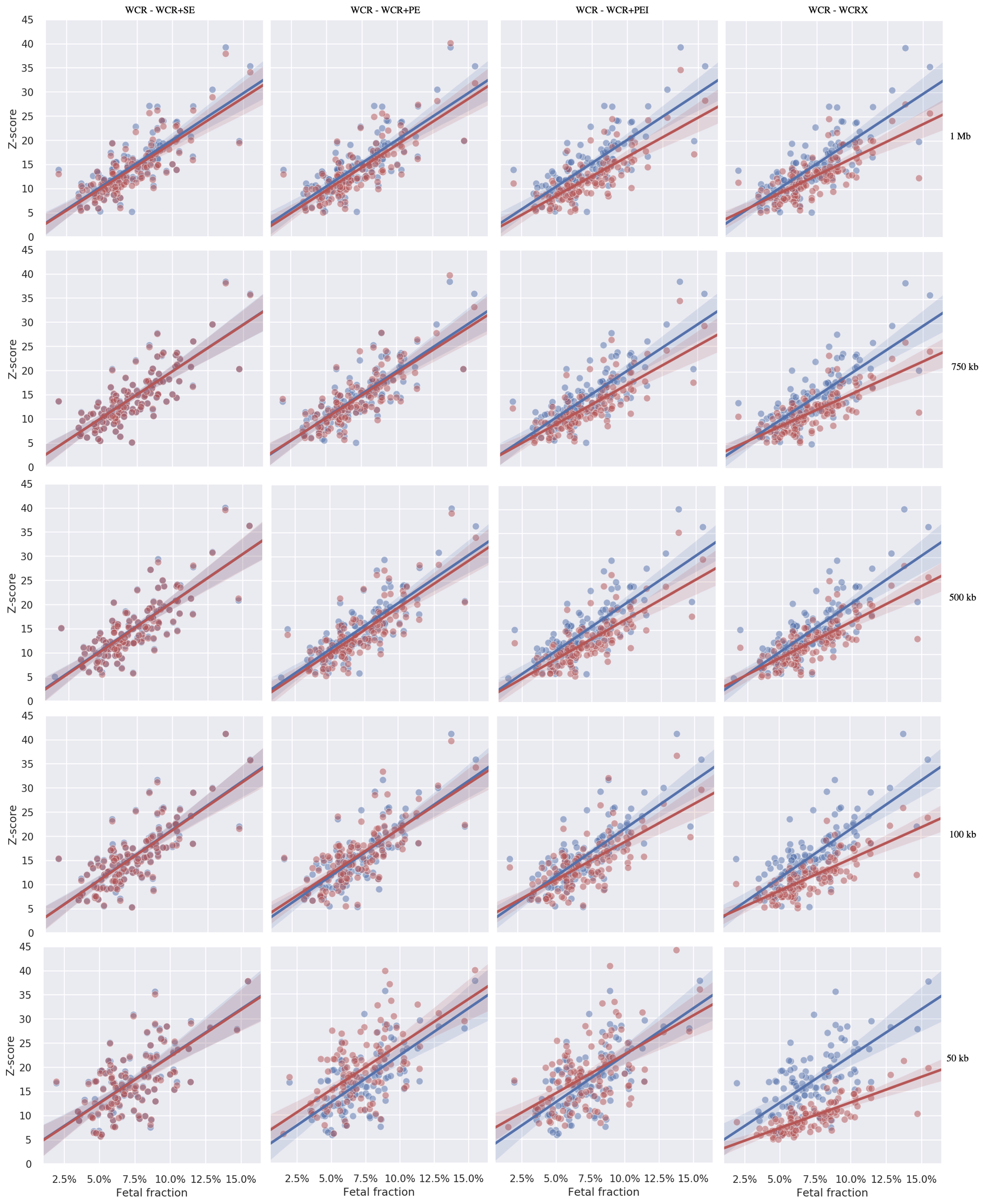

Supplement: S2 Fig — All ≥ 10 Mb events with Z-scores ≥ 5 on chromosome 21 detected by the different methods in the 125 T21 positive samples relative to the estimated fetal fractions of each sample. Each plot displays one of the methods WCR+SE, WCR+PE, WCR+PEI, and WCRX (all in red) overlaid with WCR (shown in blue), for a set bin-size resolution. Each point corresponds to a CNV within a sample. (TIF) [file pone.0284493.s002.tif]

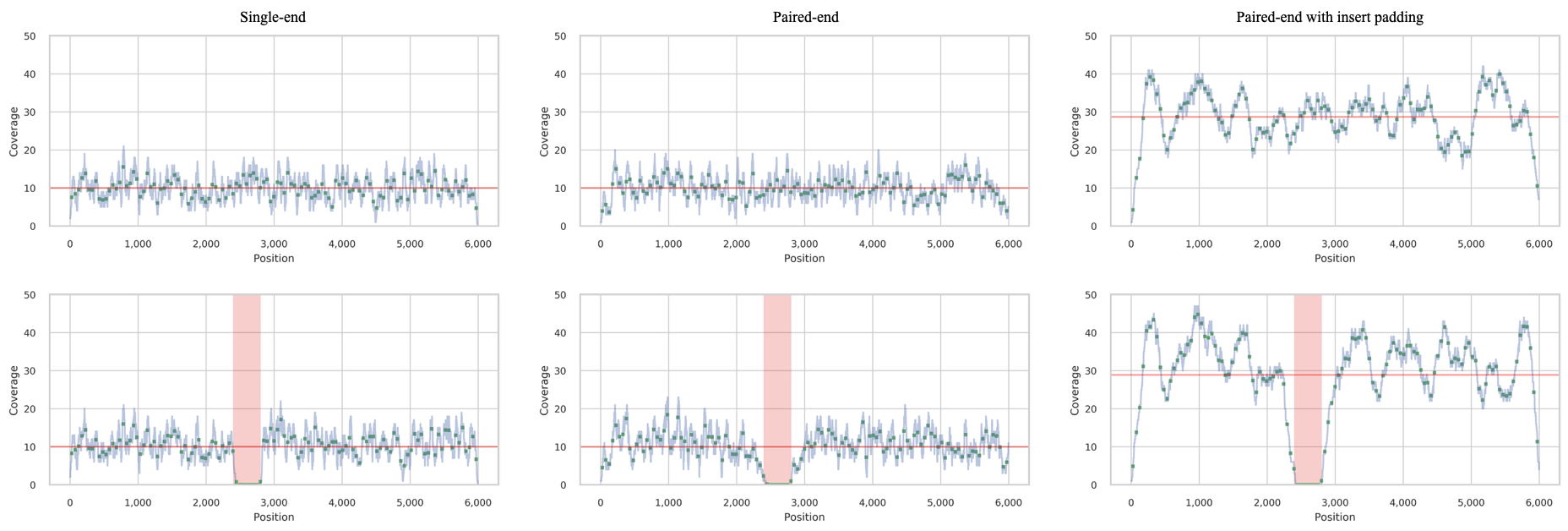

Supplement: S3 Fig — Simulated read alignment counts across a small genome of single-end, paired-end, and paired-end with insert padding methods. On top this is shown for an unaffected sample, and on the bottom for a sample with a 400 bp deletion. (TIF) [file pone.0284493.s003.tif]
