# Supplementary material for: A comprehensive performance analysis of sequence-based within-sample testing NIPT methods
Source: PLoS One. 2023 Apr 14;18(4):e0284493. doi: 10.1371/journal.pone.0284493 (PMC10104307; doi:10.1371/journal.pone.0284493)
Supplement: Attachment — Submitted filename: reviews.docx [file pone.0284493.s004.docx]

1. It has come to our attention that the manuscript seems to be a resubmission of the manuscript PCOMPBIOL-D-22-01246, which was previously considered by the journal PLOS Computational Biology. It is a requirement by the journal that any manuscript resubmitted after a previous round of review is accompanied by a rebuttal letter outlining the response to the original reviewers’ comments, so that the editors can establish whether the original concerns have been addressed. Can you therefore please provide us with a point-by-point response to the comments provided by the original reviewers, outlining the revisions carried out since the original submission.

The paper was rejected because it is not purely a **benchmark** paper. The requirements for the benchmark category in PLOS Computational Biology were not met because the paper included new additions to existing algorithms, rather than just assessing existing methods. As a consequence, no further reviews were received about the content of the paper.

2. Please provide additional details regarding participant consent. In the ethics statement in the Methods and online submission information, please ensure that you have specified what type you obtained (for instance, written or verbal, and if verbal, how it was documented and witnessed). If your study included minors, state whether you obtained consent from parents or guardians. If the need for consent was waived by the ethics committee, please include this information.

All participants of this study have given written consent. This is now correctly reflected in the manuscript, changing the Informed Consent Statement from:

“We have obtained written consent from all study participants.”

To:

 	"Written informed consent was obtained from all subjects involved in the study."
