# Supplementary material for: A comprehensive performance analysis of sequence-based within-sample testing NIPT methods
Source: PLoS One. 2023 Apr 14;18(4):e0284493. doi: 10.1371/journal.pone.0284493 (PMC10104307; doi:10.1371/journal.pone.0284493)
Supplement: Attachment — Submitted filename: rebuttal.docx [file pone.0284493.s005.docx]

Editor:

E.1. Please ensure that your manuscript meets PLOS ONE's style requirements, including those for file naming. The PLOS ONE style templates can be found at:

https://journals.plos.org/plosone/s/file?id=wjVg/PLOSOne_formatting_sample_main_body.pdf and

https://journals.plos.org/plosone/s/file?id=ba62/PLOSOne_formatting_sample_title_authors_affiliations.pdf

We have taken care to adopt the style guidelines as described above.

E.2. We note that the grant information you provided in the ‘Funding Information’ and ‘Financial Disclosure’ sections do not match. When you resubmit, please ensure that you provide the correct grant numbers for the awards you received for your study in the ‘Funding Information’ section.

We have corrected this discrepancy.

E.3. Thank you for stating the following financial disclosure:

This work is being funded by the Delft Data Science Center of the Delft University of Technology, which has no role in the design and execution of the study as well as the interpretation of the data and writing of the manuscript. Please state what role the funders took in the study. If the funders had no role, please state: "The funders had no role in study design, data collection and analysis, decision to publish, or preparation of the manuscript.". If this statement is not correct you must amend it as needed.

Please include this amended Role of Funder statement in your cover letter; we will change the online submission form on your behalf.

The statement is correct, we have amended it in the cover letter.

E.4. We note that you have stated that you will provide repository information for your data at acceptance. Should your manuscript be accepted for publication, we will hold it until you provide the relevant accession numbers or DOIs necessary to access your data. If you wish to make changes to your Data Availability statement, please describe these changes in your cover letter and we will update your Data Availability statement to reflect the information you provide.

We do not wish to change the Data Availability statement.

E.5. Your ethics statement should only appear in the Methods section of your manuscript. If your ethics statement is written in any section besides the Methods, please move it to the Methods section and delete it from any other section. Please ensure that your ethics statement is included in your manuscript, as the ethics statement entered into the online submission form will not be published alongside your manuscript.

The ethics statement was moved to the Methods section.

E.6. Please include captions for your Supporting Information files at the end of your manuscript, and update any in-text citations to match accordingly. Please see our Supporting Information guidelines for more information: <http://journals.plos.org/plosone/s/supporting-information>.

Captions of the Supporting Information files have been added to the end of the manuscript.

E.7. Please review your reference list to ensure that it is complete and correct. If you have cited papers that have been retracted, please include the rationale for doing so in the manuscript text, or remove these references and replace them with relevant current references. Any changes to the reference list should be mentioned in the rebuttal letter that accompanies your revised manuscript. If you need to cite a retracted article, indicate the article’s retracted status in the References list and also include a citation and full reference for the retraction notice.

The updated citations have been added to the cover letter.

Reviewer 1:

R1.1. The abstract and introduction talks about CNVs while the Results are mostly about Trisomy 21 test. The authors should be clear what is the focus of this work? For prenatal testing, Trisomy 21 is the most common case therefore focusing on it is acceptable.

It is accurate to state that our focus in the study was primarily on the Trisomy 21 test, as we only had access to experimental data of Trisomy 21 affected samples. However, in our simulation experiments, we thoroughly evaluated the performance of our methods in detecting copy number variations of chromosome 18, ranging from 0.25 Mb to full chromosomal deletions and duplications, at varying fetal fractions. This provides a comprehensive understanding of the performance of all methods across different scenarios and demonstrates that our testing extends beyond just Trisomy 21.

R1.2. COFFEE algorithm (Sun et al. Prenat Diagn. 2017, PMID: 28165140) is another control-free implementation for Trisomy 21 test. The authors need to discuss the differences between WISECONDOR and COFFEE.

The COFFEE algorithm is certainly interesting. It uses the difference in fragment size of the fetal and maternal DNA obtained from paired-end sequencing data to establish a partitioning of short (<150 bp) and long (>170 bp) fragments (reads between these cut-offs are excluded), wherein for each region in the genome, and for each partitioning, the read count is established and normalized. Subsequently, a paired Mann-Whitney rank-sum test is used to determine whether there is a significant change across regions. The primary novelty of COFFEE is that the fragment size and read count signals are effectively combined and that this is a control-set independent approach that does not require data from a control population. There are several significant differences relative to WISECONDOR:

1. WISECONDOR only uses the read count.

2. WISECONDOR requires a reference set of controls which are needed to create a mapping of similarly behaving bins across the genome. This mapping is what facilitates the within-sample testing.

3. WISECONDOR uses a segmentation algorithm to identify and chain regions of significant change in the read count, rather than a single region-by-region approach.

4. WISECONDOR can identify CNVs in sub-chromosomal regions.

Given these differences, we believe that the primary importance of COFFEE lies within its combined use of the fragment size and read count. Hence, we expanded the last paragraph of the discussion to detail about methods that go beyond only the read count.

**Changes in manuscript**: See R1.3.

R1.3. For PE data, do the authors need to re-mine the control regions for WISECONDOR-PE? Since fetal DNA is known to be shorter, and utilizing such information could help to improve the performance of Trisomy 21 test (Sun et al. PNAS 2018, PMID: 29760053; Chan et al. PNAS 2016, PMID: 27799561), but I did not see such implementation in WISECONDOR-PE? The authors need to discuss it.

To answer the first question. Yes, we re-mine the control regions when switching to PE data. This is specific to WISECONDOR-PE. To clarify this fact, we added the appropriate text in Methods 4.2.

The reviewer is right to point out that there is a difference in the length of fetal and maternal DNA fragments. As such there is the potential to use this as a signal to identify the absence/presence of fetal aberrations, as has been shown in both Sun et al. PNAS 2018, PMID: 29760053 and Chan et al. PNAS 2016, PMID: 27799561, as well as in Sun et al. Prenat Diagn. 2017, PMID: 28165140 (The COFFEE algorithm discussed in R1.2). We agree that the importance of this size discrepancy should be highlighted in more detail in the discussion and should refer to methods that exploit this information. On that note we also include our own contribution WisecondorFF: Diagnostics 2022 (10.3390/diagnostics12010059), which extends the within sample testing framework established by WISECONDOR to combine the read count and fragments sizes to improve CNV calling in NIPT applications.

**Changes in manuscript**:

Added the following sentence to Methods 4.2.: “*The genome-wide bin counts are unique for each Wisecondor variant, such that these counts must be generated for each*.”

Expanded the last paragraph of the discussion to: “*Possible improvements for any method utilizing paired-end reads can exploit the fact that fetal DNA fragments have a shorter fragment size than maternal fragments [31]. This can be inferred from the insert size of the aligned paired-end reads. Several methods have been proposed that leverage this fragment size difference to detect large chromosomal CNVs [32] or fetal de novo point mutations [33]. Furthermore, combining the fragment size difference with the read count signal has enabled the improved detection of fetal aneuploidies, as demonstrated by the COFFEE algorithm [34], which is a reference free method requiring no control samples. Lastly, WisecondorFF [35] was developed by our group to extend the WISECONDOR within sample testing framework and facilitate the combination of read count and fragment sizes to improve (sub)chromosomal CNV calling*.”

Added citations:

33.	Chan, K.A., Jiang, P., Sun, K., Cheng, Y.K., Tong, Y.K., Cheng, S.H., Wong, A.I., Hudecova, I., Leung, T.Y., Chiu, R.W. and Lo, Y.M.D. "Second generation noninvasive fetal genome analysis reveals de novo mutations, single-base parental inheritance, and preferred DNA ends." Proceedings of the National Academy of Sciences 113, no. 50 (2016): E8159-E8168.

34.	Yu, S.C., Chan, K.A., Zheng, Y.W., Jiang, P., Liao, G.J., Sun, H., Akolekar, R., Leung, T.Y., Go, A.T., Van Vugt, J.M. and Minekawa, R. "COFFEE: control‐free noninvasive fetal chromosomal examination using maternal plasma DNA." Prenatal diagnosis 37.4 (2017): 336-340.

35.	Mokveld, T., Al-Ars, Z., Sistermans, E.A. and Reinders, M. “WisecondorFF: Improved fetal aneuploidy detection from shallow WGS through fragment length analysis.” Diagnostics 12.1 (2022): 59.

R1.4. For Table 1, what are the specificities? Did the authors use all the control samples to build the model therefore could not calculate a specificity?

In the evaluation of the experimental data, all control samples were utilized to construct the reference sets and tested with all experimentally validated positive samples. Prior to this, in order to avoid any bias, the control samples were evaluated among themselves using smaller reference sets, such that no single control sample was tested against a reference set that included that same control sample. In these experiments no false positive calls were made on chromosome 21 on the negative control samples (data not shown). However, it should be noted that any calls made on other chromosomes could not be verified, as we only obtained experimental validation for chromosome 21. As a result, any measure of specificity would not be applicable to calls made on other chromosomes, given that no ground truth is available. Which is what necessitated the need for experiments using simulated data.

R1.5. For batch-to-batch variation, citations are needed. For instance, Sun et al. Prenat Diagn. 2017 (PMID: 28165140).

We agree with the reviewer that this is important, see R1.2 and R1.3 for details.

R.1.6. The reference list is incomplete. I saw [34, 35] in line 275, but I could not find them in Reference. Also, I did not see 31-33 in the main text.

We apologize for inadvertently omitting and miscounting some of the references in our paper and corrected this oversight.

Changes in manuscript:

Renumbered [34,35] to [31,32] and added the respective references to:

31.	Chan, K.A., Zhang, J., Hui, A.B., Wong, N., Lau, T.K., Leung, T.N., Lo, K.W., Huang, D.W. and Lo, Y.D. "Size distributions of maternal and fetal DNA in maternal plasma." Clinical chemistry 50.1 (2004): 88-92.

32.	Yu, S.C., Chan, K.A., Zheng, Y.W., Jiang, P., Liao, G.J., Sun, H., Akolekar, R., Leung, T.Y., Go, A.T., Van Vugt, J.M. and Minekawa, R. "Size-based molecular diagnostics using plasma DNA for noninvasive prenatal testing." Proceedings of the National Academy of Sciences 111.23 (2014): 8583-8588.

**Reviewer 2:**

R2.1. Line 83-85, “All samples were aligned using BWA-mem to the hg19 human reference genome with an average depth of coverage of 0.257x across all samples.” The newest released version of human reference genome is hg38. And hg38 is an improved version of hg19 with higher precision. It would be better to use newer assembly for alignment in this paper.

The reviewer has made a valid point that utilizing the latest human reference genome assembly could change the performance of CNV calling across all methods. In an ideal world, we would all be using the latest assemblies. However, in many settings, as in ours, the move towards a new assembly is not always immediately possible. This is because many downstream applications and analyses depend on specific annotations, such as gene locations and functional information, that may not yet be available for the latest assembly. This annotation dependence can make it difficult to switch to the latest assembly without first ensuring that the necessary annotations are available and have been translated to the new assembly. Note that we still utilize the hg19 human reference with WISECONDOR for our diagnostics applications given that predictions are made in conjunction with other methods, some of which only operate on hg19. Although we acknowledge the reviewer’s point, we feel that our choice to use the h19 assembly is justified, given that we are interested in finding differences between methods and not genome reference assemblies. The chosen reference should have no bearing on the comparisons that are made between the different methods, relating to different variation types, sizes, and fetal fractions, which are insightful regardless of reference basis.

R2.2. Line 166-167, “Indeed, there is a positive correlation of the fetal fraction with respect to the Z-score of the detected events for all methods.” Figure 3 showed a positive trend, but the correlation coefficient (measure the strength of a liner relationship) between the fetal fraction with the related Z-score was not described.

We agree that our wording here is too strong without providing the necessary data, Figure 3 can become more insightful by adding annotations of the R-value (and adjusted R-squared value) of the linear regression of each method, such that this statement in Results 2.4 can now be appropriately supported.

**Changes in manuscript**:

Recreated Figure 3 now with added annotations displaying the R value and adjusted R-squared value of each regression. Note that this also fixed a bug in the regression code wherein the wrong data was given in the regression of WCR+PE.

Added the sentence: “*The legend annotation relates each method with the respective R-value and adjusted R squared value of the linear fit.”* to the caption of Figure 3.

R2.3. In Figure 3, the upper two figures showed red lines while blue lines were invisible. In this case, it will be helpful if the author can state whether WCR+SE and WCR+PE (all in red) overlaid with WCR (shown in blue).

It is indeed helpful to clarify that there is no real difference in the predictions and regression when it comes to comparing WCR to WCR+SE.

Changes in manuscript:

Added the following sentence to Results 2.4. to clarify this overlap: “*With exception of WCR+SE that performs nearly identical, as can be observed from the near exact same overlapping predictions and regressions in Figure 3*.”

R2.4. In this paper, the author mainly compared the performance of T21 events. A paper published by your group “WISECONDOR: detection of fetal aberrations from shallow sequencing maternal plasma based on a within-sample comparison scheme” have presented Chromosome 19 was a difficult chromosome for WISECONDOR because of CG richness at Chromosome 19. It would be more comprehensive if other Chromosome comparisons among Wisecondor and its variants such as Chromosome 19 were included.

It would indeed be optimal to test CNV calling methods on every chromosome, including chromosome 19. In the spirit of feasibility, we opted to only test chromosomes 21 and 18. Although this approach may not provide a comprehensive evaluation of the methods across the entire genome, it is important to note that findings relating to the varying fetal fraction with respect to variation size should still be generalizable. Therefore, while our results may not be entirely representative of the performance of the methods on all chromosomes, they should still provide valuable insights into how these methods perform with respect to variation size and fetal fraction.

R2.5. In addition to the comparisons shown in the paper, one of the crucial issues for evaluating programs is runtime. Is there a significant difference in computing times between Wisecondor with its variants?

The reviewer's assertion that runtime is a crucial factor when evaluating different methods is well-founded. In our experiments, we found that there were no meaningful differences between the methods, with exception of the original legacy version of Wisecondor, which demonstrated inferior performance and an excessively long runtime in comparison to the current version available on GitHub. This inferior performance was already detailed in Results 2.1 of our study, although we had not mentioned the runtime, which we have now addressed. While WisecondorX utilizes a more optimized segmentation algorithm, namely circular binary segmentation (CBS), the benefits of this algorithm are not evident in the NIPT setting due to the relatively large size of the regions being analyzed.

Changes in manuscript:

Added the following sentence to Results 2.1. to note the poor runtime of the originally published legacy version of Wisecondor: “*Note that the originally published version of WCR (which we re-implemented) performs significantly worse than the most recent version, e.g., 118 calls at 250 kb, furthermore the run-time of this legacy implementation was at least an order of magnitude greater, hence the remaining results of this version are omitted.*”

Added the following sentence to Results 2.1 to note that there are no significant differences in runtime between the remaining methods: “*No significant differences in the run-time of the six methods were found, due to the use of relatively large bin sizes*.”
