# Supplementary material for: A comprehensive performance analysis of sequence-based within-sample testing NIPT methods
Source: PLoS One. 2023 Apr 14;18(4):e0284493. doi: 10.1371/journal.pone.0284493 (PMC10104307; doi:10.1371/journal.pone.0284493)
Supplement: Attachment — Submitted filename: rebutal.docx [file pone.0284493.s006.docx]

Reviewer 1:

R1.1. The authors had addressed most of my comments. However, I found that the citations are still inaccurate, especially the COFFFEE paper (ref. 34): the author list is completely wrong. In addition, the author had discussed and utilized fragmentation of fetal-derived cfDNA in NIPT, then they should also cite Sun et al. PNAS 2018 (PMID: 29760053) and a recent paper An et al. Nature Communications 2023 (PMID: 36653380) as these papers reveals the mechanisms to fetal DNA fragmentation for improving NIPT. The authors must take the citation seriously and correct all the errors.

We thank the reviewer for their attention to detail and apologize for the inaccuracies in our citations. We have addressed this issue and taken measures to correct this oversight. Furthermore, we have carefully examined all other citations to ensure their accuracy and completeness.

The reviewer raises a fair point that along with noting the difference in fragment size between fetal and maternal fragments, it is essential to include citations that investigate the underlying mechanisms responsible for this observed distinction.

**Changes in manuscript:**

- Citations have been corrected and renumbered
- The last paragraph has been extended to include the new citations with a short justification: *"The characteristic of fetal DNA fragments having shorter fragment size than maternal fragments is a result of the underlying mechanisms involved in DNA fragmentation, such as DNA methylation and its relation to chromatin accessibility [28,29]. The fragment size differences can be inferred from the insert size of the aligned paired-end reads."*
